# Supplementary material for: Genotype-Specific Antioxidant Responses and Assessment of Resistance Against Sclerotinia sclerotiorum Causing Sclerotinia Rot in Indian Mustard
Source: Pathogens. 2020 Oct 27;9(11):892. doi: 10.3390/pathogens9110892 (PMC7694058; doi:10.3390/pathogens9110892)
Supplement: Supplementary file 1 [file pathogens-09-00892-s001.pdf]

Supplementary table 1: Data on weather-related parameters *viz.* temperature, relative humidity and rainfall from the date of inoculation (20th January 2020) to date of last observation (06th February 2020).

**Date & month**	**Temperature (º C)**			**Relative humidity (%)**			**Rainfall (mm)**
	Maximum	Minimum	Average	Morning	Evening	Average	
20th January	9.6	6.0	7.8	100	86	93	0.0*
21th January	13.5	6.0	9.8	100	79	89	0.0
22th January	15.9	4.5	10.2	100	59	79	0.0
23th January	19.0	3.0	11.0	100	48	74	0.0
24th January	19.3	3.0	11.2	100	47	73	0.0
25th January	19.4	2.4	10.9	100	40	70	0.0
26th January	21.2	4.0	12.6	97	46	71	0.0
27th January	21.4	6.9	14.2	88	74	81	0.0
28th January	17.9	11.4	14.7	91	79	85	7.2
29th January	19.0	6.0	12.5	100	65	83	0.0
30th January	19.0	4.0	11.5	100	58	79	0.0
31th January	18.0	2.5	10.3	100	70	85	0.0
01th February	17.2	2.9	10.1	100	67	84	0.0
02th February	19.0	3.5	11.3	100	59	79	0.0
03th February	18.5	2.1	10.3	100	44	72	0.0
04th February	20.9	6.1	13.5	86	63	75	0.0
05th February	20.4	3.2	11.8	100	55	77	0.0
06th February	18.4	1.9	10.2	100	48	74	0.0
Range	9.6 - 21.4	1.9 - 11.4	7.8 - 14.7	86 - 100	40 - 86	70 - 93	0.0 - 7.2
Mean	18.2	4.4	11.3	98	60	79	0.4

Location LAT: 29° 10'N LONG: 75° 46'E, ALT: 215.2m. *Irrigation was applied just before to artificial disease inoculation to maintain the high soil moisture and 3.2 mm rainfall was also received on 14th January.
